# Evaluating the Influence of Access Cavity Design to Detect Additional Canals of Mandibular Premolars: An In Vitro Study

**DOI:** 10.1155/ijod/5575879

**Published:** 2026-04-29

**Authors:** Maryam Javidi, Ali kazemian, Mina Zarei, Hasti Erfanian Taghvaei, Melika Mohammadi

**Affiliations:** ^1^ Department of Endodontics, Faculty of Dentistry, Mashhad University of Medical Sciences, Mashhad, Iran, mums.ac.ir; ^2^ Department of Community Oral Health, Faculty of Dentistry, Mashhad University of Medical Sciences, Mashhad, Iran, mums.ac.ir; ^3^ Faculty of Dentistry, Debrecen, Hungary; ^4^ Department of Endodontics, Faculty of Dentistry, North Khorasan University of Medical Sciences, Bojnurd, Iran, nkums.ac.ir

**Keywords:** conservative access cavity, endodontic access cavity, mandibular premolar, minimally invasive treatment

## Abstract

**Objective:**

The present in vitro study aimed to investigate the impact of three different access cavity types on the identification of the additional canals in mandibular premolars.

**Methods and Materials:**

A total of 67 mandibular premolars were obtained, and periapical radiographs and CBCT scans were taken. Then, the access cavities were created in three stages: ultraconservative access cavity (UltraAC), conservative access cavity (CAC), and traditional access cavity (TAC). After completing each stage, the search for the second and third canals was conducted under a surgical microscope, using ultrasonic troughing. The differences between the three access cavity designs in identifying the second and third canals were compared using the chi‐squared test. Additionally, the agreement between periapical radiographs, CBCT, and the operator in finding the canals was determined by measuring the kappa coefficient (*p*.value = 0.05).

**Results:**

62.68%, 34.32%, and 2.98% of the samples had one, two, and three root canals, respectively. The agreement between PA radiographs and CBCT images in identifying the number of canals was almost perfect (kappa: 0.968; *p*  < 0.001). Additionally, the agreement between the operator and initial PA radiographs and between the operator and CBCT in detecting the number of canals was substantial (kappa: 0.735 and 0.794, respectively; *p*  < 0.001). No difference was observed between the access cavity types in identifying the second and third canals. Of the two three‐canal teeth, one was found in the TAC stage and the other in the CAC stage.

**Conclusion:**

When the access cavity of mandibular premolars is prepared using magnification with a microscope and ultrasonic troughing, the design of the access cavity has no effect on identifying the additional canals in these teeth.

**Clinical Relevance:**

Mandibular premolars are reported to have more than one canal, and the effect of different access cavities has not been evaluated in canal detection of these teeth.

## 1. Introduction

Appropriate access cavity preparation is a fundamental step in root canal treatment. It can promote thorough debris removal, disinfection of pulp chamber space, canal orifice detection, and effective root canal instrumentation [1, 2].

In recent years, minimally invasive dentistry is presented in order to preserve the mechanical stability of the teeth. Conservative endodontic access cavities emerged from this concept and were first proposed by Clarck and Khademi [3] in 2010.

Many studies have compared the efficacy of minimally invasive access cavity (MIAC) preparations on preservation of dentin [4], changing the geometry of root canals [5] and quality of root canal shaping, especially in molar teeth.

Fracture resistance of root canal–treated teeth is announced to be less than that of vital teeth. Precervical dentin removal and elimination of pulp chamber roof may cause the reduction of fracture resistance among root canal–treated teeth. Restorative failures are supposed to be the most relevant reason of endodontically treated teeth extractions. Therefore, access cavity preparations should be prepared as minimal as possible [6].

Mandibular premolars demonstrate great variations in root canal anatomy and may present with one, two, or three roots and canals. The prevalence of a second root canal in first and second premolars widely varies among studies (0.1%−11.2%) [7–9]. Moreover, the prevalence of the third root is reported to be 2.5% and 0.1%–3.5% in first and second premolars, respectively [7, 10].

With full knowledge of the facts, the identification of the additional canals during conservative endodontic access cavities has not been thoroughly investigated in mandibular premolars.

The aim of this study was to assess the configuration of access cavity design on the detection of additional root canals in human mandibular premolars.

## 2. Materials and Methods

Sixty‐seven extracted human mandibular premolars with full apices were used in accordance with the Mashhad Dental Faculty Ethics Committee. All mandibular premolars with one root and one or more root canals, without any calcification, caries, or restoration, resorption and crack were selected.

The sample size (*n* = 67) was determined based on the methodology used in Mendes et al. [11], who conducted a similar investigation using varying access cavity designs and visualization tools in extracted mandibular first molars. All samples were cleaned by ultrasonic scaler and kept in saline 0.9% until used. They were radiographically exposed (Nanopix Digital Sensor, Eighteeth, Changzhou, China) from two prependicular views, mesial‐distal and buccal‐lingual dimensions; then, CBCT images were prepared (Planmeca ProMax‐Xray unit, Helsinki, Finland), and anatomical configuration and number of root canals were evaluated by an endodontist.

Radiographs using paint software were cropped so that only the crown of samples was detectable, and a blind operator prepared the access cavities under magnification (12.5 ^∗^1.6) using a microscope (Zumax OMS 2380, Suzhou, China) and ultrasonic device (Ultramint pro, Eighteeth, Changzhou, China). All teeth were prepared up to three steps: ultraconservative access cavity (UltraAC), conservative access cavity (CAC), and traditional access cavity (TAC) preparation.

Because no quantitative studies have been conducted to classify UltraAC and CAC, in this study, one‐fifth of the mean bucco‐lingual width of 10 mandibular premolars (about 0.8 mm) was used as a reference to the cavity size of UltraAC.

First of all, by a high‐speed long shank round bur (Jota AG, Ruthi, Switzerland) with water spray, a notch on the occlusal surface was prepared, and then using a high‐speed fissure bur, 0.8 mm (Dia ITAL, Italy), an UltraAC was prepared, and canals were negotiated.

**Figure 1:**
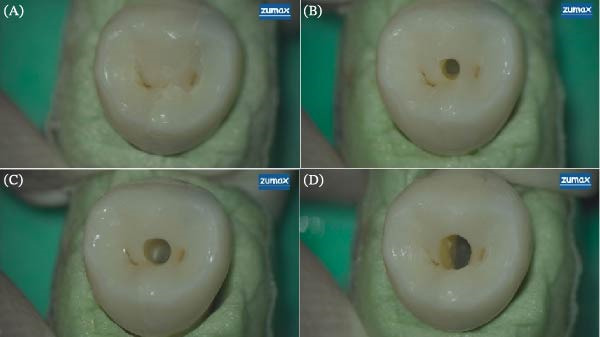
Access cavities: (A) intact teeth, (B) ultraconservative access preparation (UltraAC), (C) conservative access preparation (CAC), and (D) traditional access preparation (TAC). Magnification: 12.5 ^∗^1.6.

**Figure 2:**
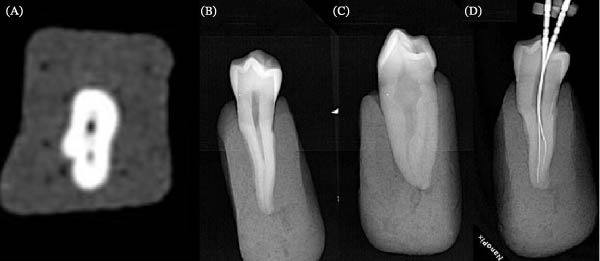
(A) CBCT axial section, (B) buccal view, (C) proximal view, and (D) canal negotiation in a mandibular two‐canal premolar.

At the second stage, in teeth where a supernumerary canal was not found, the access cavity was extended to CAC. In these samples, the access cavity was prepared by a fissure bur 1 mm bucco‐lingually at the same distance from the center of the occlusal surface. At the third stage, in teeth where an additional canal could not be found, the access cavity was changed from CAC to TAC. In order to find more canals, the pulp chamber roof was removed completely, and a straight line access to the coronal third of the root was gained (Figures 1 and 2).

After each step, irrigation with distilled water and NaOCl 2.5% (Morvabon, Tehran, Iran) by side‐vent needle 30G (Fanta irrigation needles, Shanghai, China) and endo canal suction (Arad, Biomed, Mashhad, Iran) was done. A precurved C‐pilot file ≠10 (Dentsply, Munich, Germany) and an endodontic explorer (Dental Devices, endodontic explorer, Pakistan) were used, and canals were negotiated. A gentle apical in and out motion was used, and in order to eliminate debris and better irrigation, throughing by ultrasonic E4 Tip (ultrasonic endodontic tips, Eighteeth, China) was done. Time for finding canals was set as 15 min, in each step. At last, a 20° mesial radiography was taken, and data were recorded and analyzed by SPSS (26) software; comparison of canal detection between three different access cavities was analyzed by chi‐square test (*p*.value = 0.05). The agreement between periapical radiographs, CBCT, and the operator in finding the canals was determined by measuring the kappa coefficient (*p*.value = 0.05).

## 3. Results

From 67 teeth, 42 teeth (62.68%), 23 samples (34, 32%), and two cases (2, 98%) have one, two, and three canals, respectively.

According to Cohen’s kappa coefficient, there was almost perfect agreement between conventional radiography and CBCT imaging in locating root canals (*κ* = 0.968, 95% CI: 0.909–1.027; *p*  < 0.001, Table 1).

**Table 1:** Agreement between PA radiographs and CBCT to determine the number of canals.

		Number of canals in CBCT			Total	*p*‐Value	Kappa value
		1	2	3			
Number of canals in PA	1	42	0	0	42	<0.001	0.968
	2	0	23	1	24		
	3	0	0	1	1		
	Total	42	23	2	67		

A substantial agreement was also found between clinical operation and conventional radiography, with a kappa value of 0.735 (95% CI: 0.578–0.892; *p*  < 0.001, Table 2).

**Table 2:** Agreement in PA radiographs and CBCT with operator to determine the number of canals.

Radiography	Number of canals	Number of canals found by operator			Total	*p*‐Value	Kappa value
		1	2	3			
PA	1	35	7	0	42	<0.001	0.735
	2	1	22	1	24		
	3	0	0	1	1		
	Total	36	29	2	67		
CBCT	1	35	7	0	42	<0.001	0.794
	2	1	22	0	23		
	3	0	0	2	2		
	Total	36	29	2	67		

Furthermore, the agreement between clinical operation and CBCT imaging was substantial, with *κ* = 0.794 (95% CI: 0.643–0.945; *p*  < 0.001, Table 2).

There was no significant difference between the types of access cavity preparations in detection of second and third canals of mandibular premolars. Of the two samples with three canals, one sample’s canal negotiation was done in the TAC stage and the other one, in the CAC stage (Table 3).

**Table 3:** Comparison of the number of additional canals found in different stages of access cavity preparation.

		Stage of access			Total	*p*‐Value
		UltaAC	CAC	TAC		
Number of canals found by operator	Teeth with two root canals	7	7	8	22	0.265
	Teeth with three root canals	0	1	1	2	
	Total	7	8	9	24	

## 4. Discussion

Recent studies have focused on potential impacts of conservative access cavities on fracture strength of tooth, quantifying changes in root canal configuration after instrumentation and cleaning and also the ability to locate canals [2, 12, 13].

Many researchers have investigated the effects of endodontic access cavities [4, 14, 15].

There is controversy regarding the fracture resistance of various access cavity designs in posterior teeth. Many studies have shown that there is no significant difference in fracture resistance between different access cavity designs, while some studies have reported that in the TAC design, fracture resistance is reduced compared to conservative designs [16].

There is also no consensus among studies regarding the differences between types of access cavity designs in identifying root canals in teeth with anatomical variations. Some have acknowledged that there is no difference in the detection of the mandibular mid‐mesial canals and the maxillary MB2 canals between different types of access cavities [11, 15], but another study reported that the probability of locating the MB2 canal is higher when using the CAC or TAC compared to the UltraAC (38). Other studies reported that the ability of MIACs in canal detection highly depends on the microscope and ultrasonic instruments. They concluded that with the aid of an operating microscope and ultrasonic instruments, there is not any difference in MB2 canal detection between CAC and TAC. However, this was not correct about UltraAC [17, 18].

Our study demonstrated no significant difference between the three types of access cavities in the detection of additional canals in mandibular premolars.

Almost all studies selected molar teeth for root canal locating. However, in this study, mandibular human premolar teeth were assessed. In order to the blindness of the operator, preliminary radiographs and CBCTs of samples were evaluated by an endodontist, the number of canals was detected and recorded, and a blind operator carried out the clinical procedure.

Due to the lack of precise reference for determination of access cavity dimensions in contracted access preparations, and more often UltraAC compared with CAC according to the maintenance of the roof of the pulp chamber [19], this pilot study selected five mandibular first premolars and five mandibular second premolars. The mean bucco‐lingual width was measured, and one‐fifth of that value, about 0.8 mm, was considered as UltraAC width.

In the present study, the amount of hard tissue removal in the TAC group was more than CAC, and the least was in the UltraAC group. Isufi et al. [20] found that the percentage of dentin and enamel removal was less than 6% for the UltraAC, up to 15% for the CAC, and more than 15% for the TAC group. Saygili et al. [6] evaluated the relationship between access cavity types with MB2 canal detection and found that MB2 detection was significantly higher in CAC and TAC than point access. Because there were not any differences between three types of access cavities in the detection of supernumerary canals in our study, it seems that UltraAC, which preserved more dental hard tissue, is the best choice of access cavity preparation. On the other hand, in two cases, the third canal was found in the CAC and TAC stages of preparation, and it looks like the extension of the access cavity reduced the misdetection of additional canals [15]. Although it must be kept in mind, using magnification by microscope and throughing by ultrasonic made better vision and little difference between groups and helped in locating canals in MIACs [11]. However, a sample size of more premolars with three canals is needed.

In the present study, since there were no exact references for the classification of different access cavity types, mean linear measurement was used to define UltraAC. However, future studies employing alternative measurement methods, such as a 3D volumetric approach, are recommended.

Although this study had some limitations, including in vitro design and reliance on only the Iranian population, it was the first study to evaluate different access cavity designs in detecting the second and third canals in mandibular premolars.

Our study sample size (*n* = 67) was determined based on a comparable methodological approach used in a previous anatomical study on access cavity design (Mendes et al. [11]). Given the known low prevalence of additional canals in mandibular premolars, this sample size was deemed adequate for a preliminary in vitro comparison but may limit the power to detect small differences between groups, particularly for three‐canal configurations.

A further limitation is the absence of a formal a priori power calculation. The low prevalence of multicanaled mandibular premolars increases the risk of a Type II error, particularly for three‐canal teeth (*n* = 2). Therefore, while our findings suggest no significant difference in canal detection among access designs under magnification, they should be interpreted with caution, and future studies with larger samples—especially of rare anatomical variants—are needed to confirm these results.

## 5. Conclusion

In conclusion, MIACs are useful in maintaining tooth structure. By means of microscopic magnification and ultrasonic troughing, the TAC, CAC, and UltraAC did not differ in the detection of the additional canals in mandibular premolars. However, more detailed studies with larger and ethnically diverse sample sizes are required on the endodontic access cavity effects on canal detection in mandibular premolars.

## Funding

This study is supported by the Vice Chancellor for Research of the Mashhad University of Medical Sciences, Iran (Grant 4020626).

## Ethics Statement

Human mandibular premolars, which were extracted due to orthodontic treatment or periodontal diseases, were obtained from the Oral and Maxillofacial Surgery clinic of the Mashhad Faculty of Dentistry, and patients’ written informed consents for using their extracted teeth were obtained.

## Conflicts of Interest

The authors declare no conflicts of interest.

## Data Availability

The data that support the findings of this study are available from the corresponding author upon reasonable request.
